# Bta-miR-98 Suppresses Replication of Caprine Parainfluenza Virus Type 3 Through Inhibiting Apoptosis by Targeting Caspase-3

**DOI:** 10.3389/fimmu.2020.01575

**Published:** 2020-08-28

**Authors:** Jizong Li, Chunyan Zhong, Zheng Liao, Li Mao, Wenliang Li, Min Sun, Maojun Liu, Xinqin Ji, Chuanmin Liu, Tao Xue, Leilei Yang, Wenwen Zhang

**Affiliations:** ^1^Institute of Veterinary Medicine, Jiangsu Academy of Agricultural Sciences, Key Laboratory of Veterinary Diagnosis, Key Laboratory of Veterinary Biological Engineering and Technology, Ministry of Agriculture, Nanjing, China; ^2^School of Pharmacy, Linyi University, Linyi, China; ^3^Institute of Life Sciences, School of Food and Biological Engineering, Jiangsu University, Zhenjiang, China; ^4^College of Animal Science, Guizhou University, Guiyang, China; ^5^Key Lab of Food Quality and Safety of Jiangsu Province-State Key Laboratory Breeding Base, Jiangsu Academy of Agricultural Sciences, Nanjing, China

**Keywords:** Caprine parainfluenza virus type 3, miRNA, bta-miR-98, caspase-3, apoptosis

## Abstract

Caprine parainfluenza virus type 3 (CPIV3) is an emerging respiratory pathogen that affects the sheep and goat industry in China and possibly other countries around the world. Accumulating evidence suggests that microRNAs play important roles in regulating virus-host interactions and can suppress or facilitate viral replication. In this study, we showed that CPIV3 infection induced apoptosis in Madin-Darby bovine kidney (MDBK) cells, as determined by morphological changes and flow cytometry. Caspase activity and the expression of pro-apoptotic genes further indicated that CPIV3 induced apoptosis by activating both the intrinsic and extrinsic pathways. We also demonstrated the involvement of bta-microRNA-98 (bta-miR-98) in regulating CPIV3-induced apoptosis. Bta-miR-98 was downregulated in MDBK cells infected with CPIV3. Overexpression of bta-miR-98 significantly decreased the activities of caspase-3, −8, and −9. Conversely, inhibition of bta-miR-98 had completely opposite effects. Furthermore, our data showed that bta-miR-98 markedly affected CPIV3 replication by regulating apoptosis. Importantly, we found that bta-miR-98 modulated CPIV3-induced apoptosis by targeting caspase-3, an effector of apoptosis. Collectively, our results may suggest that CPIV3 infection induced apoptosis and downregulated the levels of bta-miR-98, and this miRNA regulated viral replication through effected apoptosis. This study contributes to our understanding of the molecular mechanisms underlying CPIV3 pathogenesis.

## Introduction

Caprine parainfluenza viruses type 3 (CPIV3) belongs to the genus *Respirovirus* of the family *Paramyxiviridae*. The virus was first described in eastern China in 2014 and was found to be associated with mild to severe respiratory illness (1). The viral genome is approximately 15,618 nucleotides in length and encodes six structural proteins: nuclear protein (NP or N), phosphoprotein (P), matrix protein (M), fusion protein (F), hemagglutinin neuraminidase protein (HN), and the large polymerase unit (L). Since its discovery, CPIV3 has caught the attention of researchers in goat and sheep respiratory diseases due to its high prevalence and pathogenicity. Our previous report showed that CPIV3 antibody could be detected in 39.3% (2) and 64.9% (3) of 2,919 goat sera and 1,863 sheep sera, respectively, collected from six provinces in China. Moreover, CPIV3 genomes were frequently detected in clinical samples using real-time qPCR (4). In addition, the pathogenicity of CPIV3 in goats was systematically evaluated in our previous study (5). Infection caused cough, nasal discharge, dyspnea, viremia, virus shedding, and lung consolidation. Moreover, horizontal transmission of the virus in goats was confirmed, as shown by the presence of obvious disease characteristics (5). However, few studies have addressed the replication mechanisms and pathogenesis of CPIV3.

Apoptosis is one of the main pathways of programmed cell death following the activation of an intracellular self-destructive biochemical program (6, 7). Two distinct signaling pathways are involved in apoptosis. The extrinsic signaling pathway is triggered by activation of transmembrane death receptors, which mediates subsequent apoptotic signal transduction and results in the activation of caspase-8. Caspase-8 activates caspase-3 and caspase-7, leading to apoptosis (8, 9). In contrast, the intrinsic signaling pathway is controlled by mitochondria (10, 11). Cytochrome c released from mitochondria leads to the activation of caspase-9, and then caspase-3 is recruited by caspase-9 to cleave many cellular substrates, thereby triggering apoptosis (12). Thus, caspase cascades that are activated by both extrinsic and intrinsic signaling lead to cell death. Accumulating evidence has shown that apoptosis induced by virus infection can have both negative and positive effects on viral replication. On the one hand, host cells eliminate infected cells through apoptosis, reducing virus propagation (13, 14). On the other hand, a number of viruses may enhance host cell viability to prolong the release of virus particles (10, 15). Recent studies revealed that apoptosis was induced in cultured and primary cells by some members of the family *Paramyxiviridae* such as Newcastle disease virus (16, 17), Sendai virus (18, 19), canine distemper virus (20, 21), and *peste des petits ruminants virus* (22). The apoptotic mechanisms of these viruses were further evaluated. Herein, we wanted to understand how CPIV3 triggered apoptosis signaling pathways and the relationships between apoptosis and CPIV3 replication.

MicroRNAs (miRNAs) are a class of small, endogenous, non-coding RNAs, ∼22 nucleotides in length, which are incorporated into the RNA-induced silencing complex, including members of the Argonaute family of proteins (23). The complex is responsible for the regulatory functions of miRNAs, which lead to translational repression or degradation of target mRNAs (24, 25). Growing evidence suggests that miRNAs act as key regulators of a wide variety of biological processes including cell growth, differentiation, and apoptosis under both physiological conditions and disease states such as viral infection (26, 27). Numerous studies have documented the roles of miRNAs in the regulation of apoptosis, and several of these miRNAs exerted their effects by directly targeting genes involved in caspase cascades. For example, miR-23a and miR-24a regulated mitochondria-mediated apoptosis by inhibiting the expression of caspase-9 (28, 29). Hudson et al. (30) showed that miR-106b-25 was upregulated in human prostate cancer and directly targeted caspase-7 mRNA to modulate apoptosis. The executioner caspase-3 was a target of miR-421 (31), miR-528-5p, and miR-363 (32), and these miRNAs were demonstrated to inhibit apoptosis in cell cultures. However, data are still lacking regarding miRNA regulation of CPIV3 infection, and specifically regarding the role of miRNAs in viral regulation of apoptosis. Our previous work on CPIV3-infected MDBK cells showed that dysregulated miRNAs were involved in the regulation of apoptosis, signal transduction, and immune responses against CPIV3 (33). Thus, miRNAs may be important for the regulation of apoptotic signaling pathways in CPIV3-infected MDBK cells.

In this study, MDBK cells were infected with CPIV3. The molecular and morphological characteristics of CPIV3-infected cells showed that CPIV3 induced apoptosis and appeared to activate both caspase-8 and caspase-9 via extrinsic and intrinsic signaling pathways. In addition, we further demonstrated that bta-miR-98 suppressed CPIV3 replication and inhibited apoptosis via targeting caspase-3. These data suggest a regulatory role of apoptosis during CPIV3 replication in which bta-miR-98 influences CPIV3-induced apoptosis.

## Materials and Methods

### Cells and Viruses

MDBK and HEK-293T cells were purchased from the China Institute of Veterinary Drug Control. Cells were cultured in Dulbecco’s modified Eagle medium (DMEM; Sigma, United States) supplemented with 10% fetal bovine serum (FBS; HyClone, United States), 100 mg/mL penicillin, and 100 IU/mL streptomycin. The cultures were incubated at 37°C in a humidified incubator under an atmosphere containing 5% CO_2_.

CPIV3 JS2013 strain (GenBank accession no. NC_028362.1) was isolated in Jiangsu Province, China, and was stored in our laboratory (1). A viral titer of 10^7^.^0^ 50% tissue culture infective dose (TCID_50_)_/_mL was determined using the Reed-Muench method (34).

### Morphological Observations

MDBK cells were seeded in six-well plates and incubated with CPIV3 strain JS2013 at a multiplicity of infection (MOI) of 1. After 1 h, the cells were cultured in DMEM with 2% FBS. Uninfected cells were included as controls. Each experiment was performed in triplicate. Morphological changes were observed using a fluorescence microscope (Olympus, IX51, Japan).

### Flow Cytometry

Apoptosis in CPIV3-infected MDBK cells at different time points was assessed using an annexin V-fluorescein isothiocyanate (FITC) apoptosis detection kit (Beyotime, Shanghai, China) following the manufacturer’s instructions. Briefly, MDBK cells were harvested and washed three times with phosphate-buffered saline (PBS). After washing, cells were incubated with 5 μL of FITC-conjugated annexin V antibody and 5 μL of propidium iodide (PI) in 500 μL of binding buffer at room temperature for 15 min. Data were acquired using a flow cytometer (BD Biosciences, San Jose, CA, United States).

### Caspase Activity Assay

The activities of caspase-3, -8, and -9 were measured using a colorimetric assay kit (Beyotime, Shanghai, China) according to the manufacturer’s recommendations. Briefly, cells were harvested and treated with 100 μL of lysis buffer for 15 min. Subsequently, the protein concentrations were measured using bicinchoninic acid protein assay reagent (Beyotime, Shanghai, China). Then, 150 μg of each lysate were incubated with 10 μL of caspase−3, −8, and −9 substrate (2 mM) at 37°C for 4 h. Absorbance values were measured at 405 nm using a microplate spectrophotometer (BioTek, FLx800, United States).

### Quantitative Reverse Transcriptase PCR

To assess apoptosis-regulating gene expression, total RNA was isolated from each sample using TransZol UP reagent (Transgen Co., Ltd., Beijing, China). PrimeScript^TM^ RT Master Mix (TaKaRa, Dalian, China) was used to synthesize first-strand cDNA. Quantitative reverse transcriptase PCR (qRT-PCR) was performed using a SYBR Premix Ex Taq^TM^ kit (TaKaRa, Dalian, China) according to the manufacturer’s instructions. The specific primers used to quantitate expression of apoptosis-regulating genes are listed in Table 1. The primers were designed using Primer Premier 5.0 software. GAPDH was used as a reference gene to normalize the quantification cycle (Cq) values of other products. The parameters for amplification were as follows: initial preincubation at 95°C for 30 s, followed by 40 cycles each consisting of 5 s at 95°C and 34 s at 60°C. Three independent biological replicates were performed for each gene. Expression fold changes for target transcripts were calculated using the 2^–ΔΔcq^ method.

**TABLE 1:** Sequences of primers, miRNAs, and siRNAs used in this study.

Name	Sequence (5′-3′)	Application	*T* (°C)
qmiR-98-F	TGAGGTAGTAAGTTGTATTGTT	Real-time PCR detection of bta-miR-98	60
5S rRNA-F	GTCTACGGCCATACCACCCT	Real-time PCR detection of 5S rRNA	60
qCPIV3F	GCTTGGCTTCTTTGAAATGG	Real-time PCR detection of CPIV3 JS2013	60
qCPIV3R	GCCTGCAGAAGTTCCTTGTC		
qCPIV3-probe	FAM-CAATCGGACTAGCCAAGTATGGTGGGA-TAMRA		
qcaspase-3-F	TGGTGCTGAGGATGAC	Real-time PCR detection of caspase-3	60
qcaspase-3-R	AAAGAGCCTGGATGAA		
qcaspase-8-F	AGTGCCCTTCCCTTATTGGC	Real-time PCR detection of caspase-8	60
qcaspase-8-R	CAGGCCCCATTCCAGATGTT		
qcaspase-9-F	GATCAGGCCAGGCAGCTAAT	Real-time PCR detection of caspase-9	60
qcaspase-9-R	CGGCTTTGATGGGTCATCCT		
qFas-F	ACCCGGAATACCAAGTGCAG	Real-time PCR detection of Fas	60
qFas-R	GTTGCTCGTTGGTGTGCATT		
qFasL-F	GCCTCCACACGACTGAAGAA	Real-time PCR detection of FasL	60
qFasL-R	GTCCACCCAGAAGATTGGGG		
qBax-F	GCCCTTTTGCTTCAGGGTTT	Real-time PCR detection of Bax	60
qBax-R	ACAGCTGCGATCATCCTCTG		
qBcl-2-F	GGGGTCATGTGTGTGGAGAG	Real-time PCR detection of Bcl-2	60
qBcl-2-R	CAGACTGAGCAGTGCCTTCA		
GAPDH-F	GATTGTCAGCAATGCCTCCT	Real-time PCR detection of GAPDH	60
GAPDH-R	GGTCATAAGTCCCTCCACGA		
caspase-3-3′UTR-F	GGGGTACCCCATATGAGTCCTTTTCTACCG *Kpn* I	Cloning caspase-3 3′UTR	65
caspase-3-3′UTR-R	GAAGATCTTCCATATCGTCATTGCTGTTGT *Bgl* II		
caspase-8-3′UTR-F	GGGGTACCCCAGCCTCAGCAATCCGACGTG *Kpn* I	Cloning caspase-8 3′UTR	65
caspase-8-3′UTR-R	GAAGATCTTGCTTTTCAAGTGTTAGGAGA *Bgl* II		
caspase-9-3′UTR-F	GGGGTACCCCACGCTGCCTGCAGTGACATT *Kpn* I	Cloning caspase-9 3′UTR	65
caspase-9-3′UTR-R	GAAGATCTTCTGTTTGTAAATTCCTTTCA *Bgl* II		
bta-miR-98	UGAGGUAGUAAGUUGUAUUGUU	Overexpression bta-miR-98	−
bta-miR-98 inhibitors	AACAAUACAACUUACUACCUCA	Inhibition of bta-miR-98	−
bta-miR-98-mut	UCUCCAUCUAAGUUGUAUUGUU	Mutation of bta-miR-98	−
NC	UUCUCCGAACGUGUCACGUTT	Negative control	−
NC inhibitor	CAGUACUUUUGUGUAGUACAA		
caspase-3-309	GCAGCAAACCUCAGGGAAATT	RNA interference	−
caspase-3-487	CCAACGGACCCGUCAAUUUTT		
caspase-3-828	GCAAUAGAAUAUGAGUCCUTT		

To quantify the expression of mature miRNAs, these miRNAs were isolated using the miRcute miRNA isolation kit (TIANGEN, Beijing, China), and cDNA was prepared using a poly(dT) primer (TIANGEN, Beijing, China). qPCR was performed using a specific bta-miR-98 primer (Table 1) and a miRcute miRNA qPCR Detection Kit (TIANGEN, Beijing, China) according to the manufacturer’s instructions. Samples were heated at 95°C for 2 min, followed by 40 cycles of 95°C for 20 s and 60°C for 34 s. Relative expression levels of bta-miR-98 were normalized to levels of the internal standard control 5S rRNA within each sample using the 2^–Δ^ ^Δ^ ^*cq*^ method.

The amount of CPIV3 RNA was quantitated by qRT-PCR as previously described (4). Briefly, a plasmid containing a 150 bp fragment of the CPIV3 M gene was used to produce a standard curve. Total RNA was isolated from each sample using TransZol UP reagent. The extracted RNA was used for quantification of CPIV3 genomic RNA by qRT-PCR using specific primers and probes, listed in Table 1. The reaction was performed using an ABI Step One Real-Time PCR System (ABI, Sacramento, CA, United States) under the following conditions: 42°C for 5 min, 95°C for 10 s, followed by 40 cycles of 95°C for 5 s and 60°C for 34 s. A CPIV3-containing sample was used as a positive control and PCR-grade water was used as a negative control.

### Western Blotting

Cells were treated as indicated and then washed with PBS, lysed in Tris-lysis buffer (Beyotime, Shanghai, China) in an ice bath for 20 min, and centrifuged (12,000 × *g*, 10 min). The supernatant was mixed with 6 × SDS loading buffer, then boiled for 10 min, separated by SDS-PAGE, and transferred to nitrocellulose membranes. The membranes were blocked with 5% non-fat milk at 4°C overnight and incubated with different primary antibodies [rabbit anti-caspase-3, -8, and -9 polyclonal antibody (Abcam, United States) or anti-β-actin monoclonal antibody (Beyotime, Shanghai, China), all antibodies were diluted 1:1000] at 37°C for 2 h. The membranes were then washed with PBS containing 0.05% Tween-20 and reacted with corresponding horseradish peroxidase-conjugated secondary antibodies at 37°C for 1 h. All blots were developed using enhanced chemiluminescence reagents (Vazyme, Nanjing, China).

### miRNA Overexpression and Inhibition in MDBK Cells

All bta-miR-98 mimics and inhibitors, including the corresponding negative control (NC) molecules, were synthesized by GenePharma Co., Ltd. (Shanghai, China). MDBK cells were transfected with bta-miR-98 mimics and inhibitors using Xfect^TM^ MicroRNA Transfection Reagent (TaKaRa, Dalian, TAKARA) according to the manufacturer’s recommendations. Twenty-four hours post-transfection, cells were harvested and qRT-PCR was performed to quantitate bta-miR-98 expression. In another group of experiments, cells were infected with CPIV3 at an MOI of 0.01 for 24 and 48 h. The cells and culture supernatants were harvested to determine viral titers.

### Viral Titration

Production of viral progeny was assessed using TCID_50_ assays as described previously (35). MDBK cells were trypsinized and seeded in 96-well plates 24 h prior to infection. Virus supernatants were serially diluted 10-fold and added to each well in eight replicates. Four days post-infection, cells showing cytopathic effect (CPE) were scored positive for viral infection and the TCID_50_ was calculated by the Reed-Muench method (34).

### Dual-Luciferase Reporter Assay

The partial 3′-UTR gene of caspase-3, containing the predicted bta-miR-98 binding site (5′-CUACCUCA-3′) and the complete 3′-UTR gene of caspase-8, 9 was amplified by PCR and cloned into a pGL3-control plasmid (Promega) between the *Kpn*I and *Bgl*II restriction sites. For the reporter assays, HEK-293T cells were seeded in 24-well plates prior to transfection, then transfected with the luciferase reporter plasmid, pRL-CMV plasmid (Promega) encoding Renilla luciferase, and either bta-miR-98 mimics, inhibitors, or NC molecules using Lipofectamine 2000 (Invitrogen, United States) according to the manufacturer’s recommendations. Forty-eight hours later, firefly and Renilla luciferase activities were measured using a dual-luciferase reporter assay kit (Promega). Each experiment was performed three times, and the relative expression levels of firefly luciferase activity were normalized to Renilla luciferase activity.

### RNA Interference

Small interfering RNAs (siRNAs) specific for caspase-3 mRNA were synthesized by GenePharma Co., Ltd. (Shanghai, China). Cells were transfected with 10 nM of each siRNA or NC miRNA using Lipofectamine 2000 (Invitrogen, United States). Forty-eight hours post-transfection, cells were harvested and qRT-PCR and western blotting were performed to assess caspase-3 mRNA expression. In another group of experiments, cells were infected with CPIV3 at an MOI of 0.01. Cells and culture supernatants were harvested to determine viral titers.

### Statistical Analyses

Statistical analyses were conducted using SPSS 24.0 (version 13.0). Student’s *t*-tests and analysis of variance were used to assess differences between two groups and among multiple groups, respectively. Graphs were prepared using GraphPad Prism (GraphPad software, La Jolla, CA, United States). In all figures, data were expressed as means ± standard deviations (SDs) or standard errors of the means (SEMs) of three independent experiments. Differences were considered statistically significant at *P* < 0.05 (^∗^) and highly significant at *P* < 0.01 (^∗∗^).

## Results

### CPIV3 Infection Induced Time-Dependent Apoptosis in MDBK Cells

**FIGURE 1:**
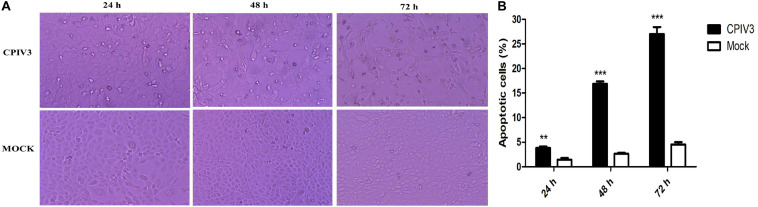
CPIV3 infection induced apoptosis in MDBK cells. **(A)** Morphological changes in CPIV3- and mock-infected MDBK cells at 24, 48, and 72 h (magnification, ×100). **(B)** MDBK cells were infected with CPIV3 (MOI = 1) at 24, 48, and 72 h. The cells were dual-labeled with annexin V and PI and analyzed by flow cytometry. **(C)** The proportions of apoptotic cells among the total cells were expressed as the means ± SEMs of three independent experiments (**P < 0.01; ***P < 0.001).

After infection with CPIV3 at an MOI of 1, morphological changes were observed in MDBK cells. Cell rounding and improved refractivity were visible at 24 h post-infection (hpi), and more evident at 48 hpi. Most cells were detached from the culture surface at 72 hpi when compared with mock-infected MDBK cells (Figure 1A). Progression of CPIV3-induced apoptosis was assessed by flow cytometry. Apoptosis significantly increased at 24 hpi, and the number of apoptotic cells increased with infection time, reaching about 27% at 72 hpi (Figure 1B). No obvious signs of apoptosis were observed in mock-infected cells. Our findings suggested that apoptosis was induced by CPIV3 infection in MDBK cells in a time-dependent manner.

### CPIV3-Induced Apoptosis Was Activated via Both Extrinsic and Intrinsic Signaling Pathways

**FIGURE 2:**
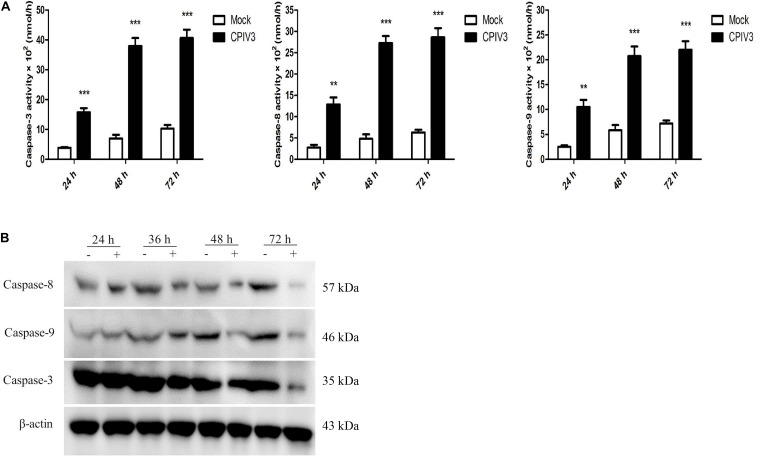
Expression levels of caspase-3, -8, and -9 in CPIV3-infected MDBK cells. **(A)** The activities of caspase-3, -8, and -9 in MDBK cells infected with CPIV3 (MOI = 1) at designated times points were tested using colorimetric assay kits. The results are presented as the means ± SEMs (**P < 0.01; ***P < 0.001). **(B)** Western blotting to assess caspase-3, -8, and -9 expression levels in MDBK cells infected with CPIV3 at the designated time points.

We next investigated the mechanisms of apoptosis induced by CPIV3 infection in MDBK cells. The activities of caspase-3, -8, and -9 were assessed by colorimetric analysis and western blotting. Expression levels of caspase-3, -8, and -9 were significantly increased at 24 hpi, and then increased further over time until 72 hpi in CPIV3-infected cells compared with mock-infected cells (Figure 2A). Western blotting results were consistent with those of colorimetric analysis (Figure 2B), suggesting that CPIV3-induced apoptosis may be triggered through both extrinsic and intrinsic pathways.

### Apoptosis Was Regulated by Fas and Bcl-2 Family Members

**FIGURE 3:**
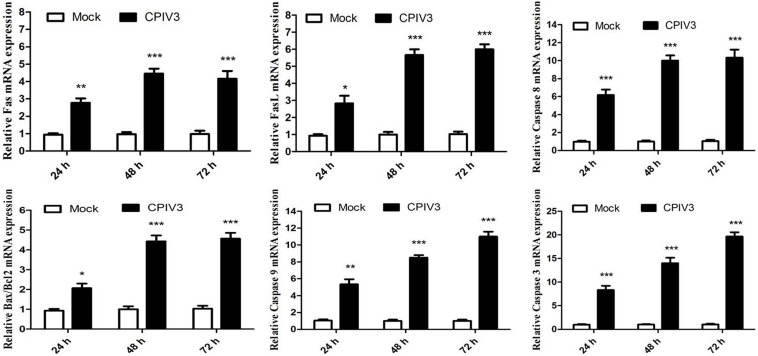
CPIV3-induced apoptosis was trigged by the expression of Fas/FasL and members of the Bcl-2 family. The expression levels of Fas/FasL, caspase-8, Bcl-2, Bax, and caspase-9 were analyzed by qRT-PCR post-CPIV3 infection. The data are shown as means ± SEMs (*P < 0.05; **P < 0.01; ***P < 0.001). Each experiment was performed in triplicate.

Caspase-8 plays an essential role in apoptosis mediated by the Fas/FasL family. Caspase-8 expression was significantly increased in CPIV3-infected MDBK cells, indicating that CPIV3 induced apoptosis via the Fas/FasL pathway. We quantified the expression levels of Fas/FasL and caspase-8 in CPIV3-infected cells by qRT-PCR and found that their expression increased over time (Figure 3). To determine whether activated caspase-9 modulated apoptosis via mitochondrial pathways, expression levels of Bcl-2 and Bcl-2-associated X protein (Bax) were assessed by qRT-PCR in CPIV3-infected cells. Levels of caspase-3 and caspase-9 mRNA and the Bax: Bcl-2 ratio markedly increased starting from 24 hpi and continued to increase until 72 hpi (Figure 3). These results indicated that Fas/FasL might play essential roles in regulating the activation of caspase-8, and that Bax- and Bcl-2-mediated signaling contributed to activation of caspase-9, eventually resulting in activation of caspase-3.

### Downregulation of bta-miR-98 During CPIV3 Infection

**FIGURE 4:**
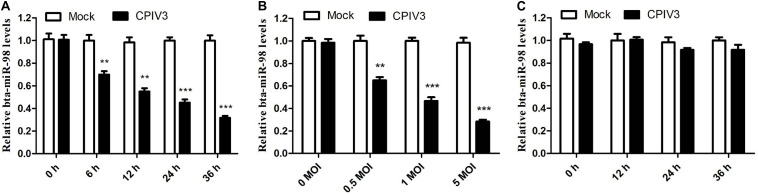
Bta-miR-98 expression was downregulated during CPIV3 infection. **(A,B)** MDBK cells were infected with CPIV3 for the indicated durations **(A)** or at the indicated MOIs for 24 h **(B)**. **(C)** MDBK cells were infected with UV-inactivated CPIV3 (MOI = 1) for 24 h (**P < 0.01; ***P < 0.001).

In our previous study, we showed through deep sequencing that bta-miR-98 was one of the most significantly downregulated miRNAs in CPIV3-infected MDBK cells (33). Because bta-miR-98 was involved in regulating apoptotic pathways, it was selected for further analysis in relation to CPIV3 infection. The expression patterns of bta-miR-98 were evaluated by qRT-PCR. Bta-miR-98 was significantly downregulated in both a time- (Figure 4A) and dose-dependent (Figure 4B) manner. By contrast, UV-inactivated CPIV3 failed to induce bta-miR-98 downregulation in MDBK cells (Figure 4C). These results revealed that bta-miR-98 was strongly downregulated during CPIV3 infection.

### Effects of bta-miR-98 on CPIV3-Induced Apoptosis

**FIGURE 5:**
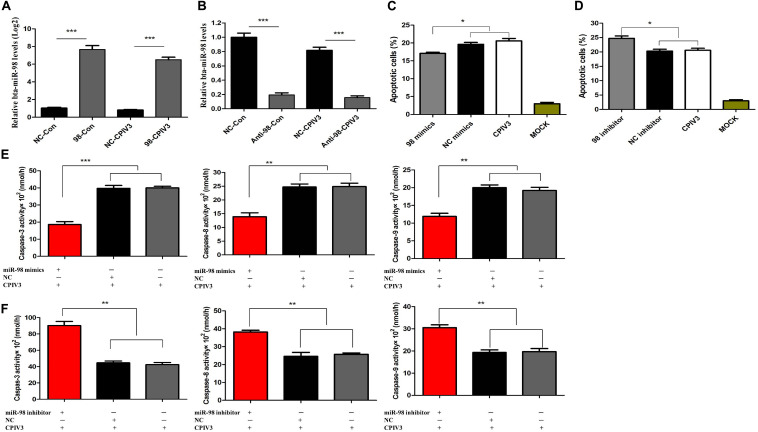
Bta-miR-98 reduced CPIV3-induced apoptosis. **(A,B)** MDBK cells were transfected with bta-miR-98 mimics, inhibitors, or NCs for 24 h and then either infected or mock-infected with CPIV3 (MOI = 1) for 24 h. Levels of bta-miR-98 were determined by qRT-PCR and normalized to levels of 5s rRNA. **(C,D)** MDBK cells were transfected with bta-miR-98 mimics, inhibitors, and NCs for 24 h and infected with CPIV3 (MOI = 1) for 48 h. The cells were dual-labeled with annexin V and PI and analyzed by flow cytometry. The proportions of apoptotic cells among total cells were expressed as means ± SEMs of results from three independent experiments. **(E,F)** MDBK cells were transfected with bta-miR-98 mimics, inhibitors, and NCs for 24 h and infected with CPIV3 (MOI = 1) for 48 h. The activity of caspase-3 in MDBK cells was tested using colorimetric assay kits. The results are presented as the means ± SEMs (*P < 0.05; **P < 0.01; ***P < 0.001).

We showed that CPIV3 infection could produce CPE and apoptosis in MDBK cells, and that bta-miR-98 was downregulated during CPIV3 infection. To assess whether bta-miR-98 was involved in CPIV3-induced apoptosis, the ability of bta-miR-98 to regulate caspase activity was determined. As shown in Figures 5A,B, bta-miR-98 mimics effectively increased endogenous bta-miR-98 levels in both mock- and CPIV3-infected cells at 24 hpi, whereas bta-miR-98 inhibitors reduced it. Interestingly, following transfection with bta-miR-98 mimics, the percentage of apoptotic cells was significantly decreased (*P* < 0.05) and the activities of caspase-3, -8, and -9 were significantly downregulated (Figures 5C,E). In contrast, inhibition of bta-miR-98 promoted CPIV3-induced apoptosis and increased caspase activation (Figures 5D,F). Together, these results indicated that bta-miR-98 participates in regulating CPIV3-mediated apoptosis.

### Bta-miR-98 Inhibits CPIV3 Replication

**FIGURE 6:**
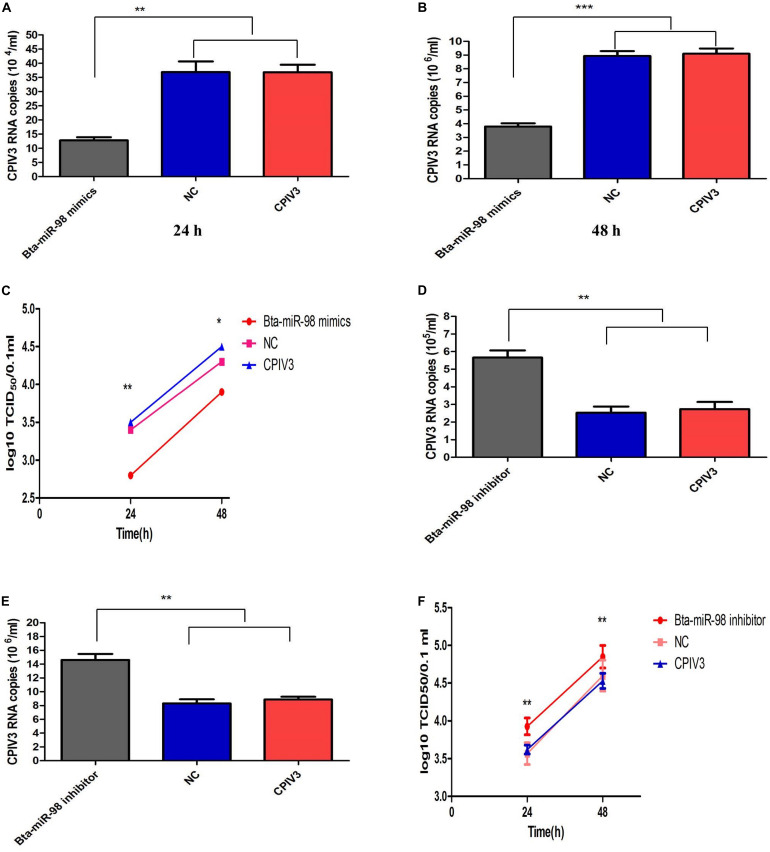
Bta-miR-98 reduced replication of CPIV3. MDBK cells were transfected with bta-miR-98 mimics, inhibitors, and NCs for 24 h and infected with CPIV3 (MOI = 0.01) for 24 and 48 h. CPIV3 replication was evaluated by qRT-PCR **(A,B,D,E)**, and TCID_50_ assay **(C,F)**. Results are representative of three independent experiments and are presented as means ± SDs (*P < 0.05; **P < 0.01; ***P < 0.001).

To confirm the effects of bta-miR-98 on CPIV3 replication, MDBK cells were transfected with bta-miR-98 mimics, inhibitors, or corresponding control miRNAs and then infected with CPIV3 at an MOI of 0.01. Culture supernatants from infected cells were harvested at 24 and 48 hpi and subjected to virus titration and qRT-PCR analysis. As shown in Figures 6A–C, CPIV3 mRNA levels were significantly reduced. Moreover, viral titers of CPIV3 were markedly decreased cells transfected with bta-miR-98 mimics compared with cells transfected with NC molecules at 24 and 48 hpi. By contrast, knockdown of bta-miR-98 significantly promoted CPIV3 replication (Figures 6D–F). These data suggested that bta-miR-98 acts as an antiviral factor during CPIV3 infection.

### Caspase-3 Is a Potential Target of bta-miR-98 During CPIV3 Infection

**FIGURE 7:**
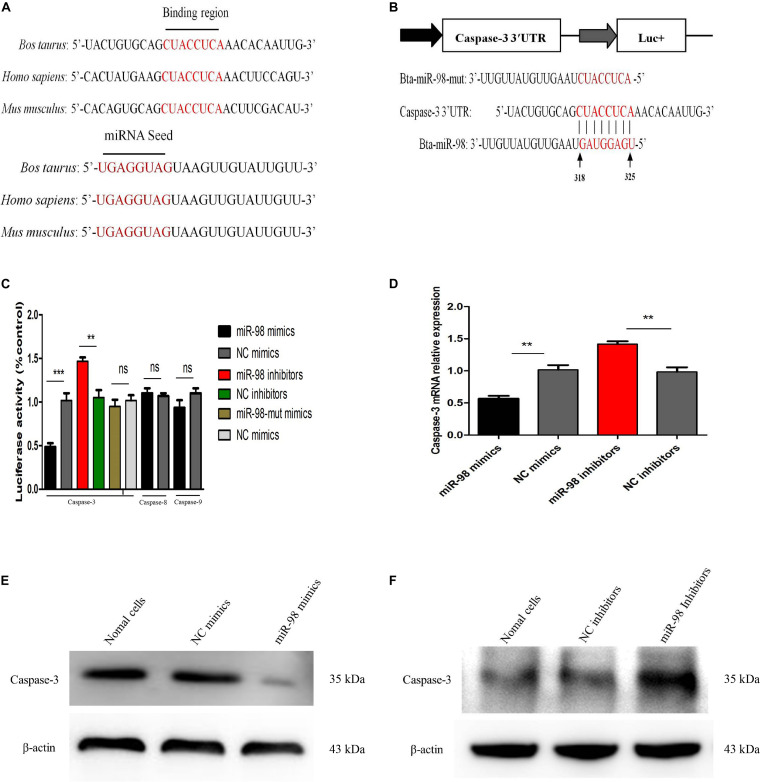
Bta-miR-98 directly targets caspase-3. **(A)** Conservation of the sequence of bta-miR-98 (lower panel) and its target sequence in caspase-3 (upper panel) among different species. **(B)** Dual-luciferase reporter construct carrying the caspase-3 3′UTR with putative bta-miR-98 binding site. The lower panel shows the predicted target sites for bta-miR-98 in the caspase-3 3′UTR. Seed regions and mutated bta-miR-98 are colored red. **(C)** 293T cells were co-transfected with bta-miR-98 mimics, bta-miR-98 inhibitors, bta-miR-98-mut mimics, or corresponding control miRNAs together with the caspase-3, -8, and -9 3′UTR luciferase reporter plasmid and pRL-CMV plasmid (Renilla luciferase activity). Firefly luciferase activities were measured and normalized to Renilla luciferase activities after 48 h. **(D–F)** MDBK cells were transfected with bta-miR-98 mimics, bta-miR-98 inhibitors, or corresponding control miRNAs. At 24 h post-transfection, caspase-3 mRNA and protein levels were assessed by qRT-PCR and western blotting, respectively. Results are representative of three independent experiments and presented as means ± SDs (**P < 0.01; ***P < 0.001).

We had established that caspase-3 played an important role in CPIV3-induced apoptosis. The potential targets of bta-miR-98 were predicted using TargetScan and miRanda software. The caspase-3 3′ untranslated region (UTR) contained a conserved bta-miR-98 8-mer seed match sequence at positions 318–325 (Figure 7B). The sequences of the bta-miR-98 and caspase-3 3′UTRs were compared with those of different species, and were highly conserved (Figure 7A). To determine whether bta-miR-98 binds to the caspase-3 3′UTR, a dual-luciferase reporter assay was performed using a plasmid containing a putative binding site for bta-miR-98. Luciferase activity was significantly reduced in 293T cells co-transfected with bta-miR-98 mimics and a caspase-3 3′UTR bearing a bta-miR-98 binding site, while markedly increased luciferase activity was detected following application of bta-miR-98 inhibitors (Figure 7C). Bta-miR-98-mut alone had no effect on luciferase activity (Figure 7C). Moreover, Luciferase activity was not changed when co-transfected with bta-miR-98 mimics and caspase-8, -9 3′UTR (Figure 7C).

To further evaluate whether caspase-3 was a potential bta-miR-98 target, expression of caspase-3 was assessed in MDBK cells transfected with bta-miR-98 mimics or inhibitors. Overexpression of bta-miR-98 reduced the expression of caspase-3 at both the mRNA and protein levels, whereas bta-miR-98 inhibitors restored caspase-3 expression (Figures 7D–F). These data indicated that caspase-3 was targeted and regulated by bta-miR-98.

### Bta-miR-98 Suppresses Replication of CPIV3 by Inhibiting Apoptosis and Targeting Caspase-3

**FIGURE 8:**
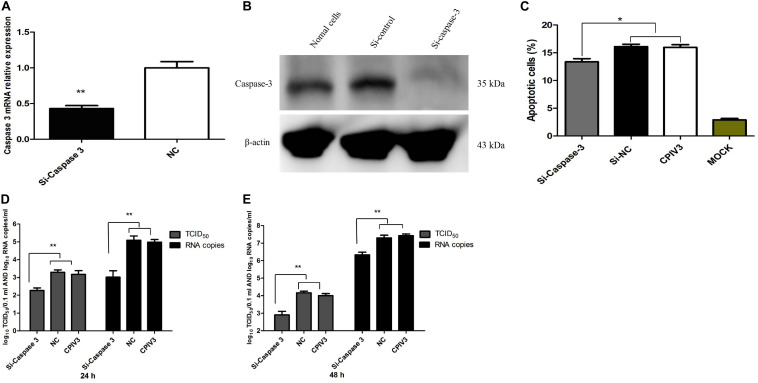
Silencing of caspase-3 inhibited apoptosis of MDBK cells and suppressed CPIV3 replication. **(A,B)** MDBK cells were transfected with siRNA or siRNA control for 24 h, and then caspase-3 mRNA and protein levels were assessed by qRT-PCR and western blotting, respectively. **(C)** MDBK cells were transfected with siRNA or siRNA control for 24 h and then infected with CPIV3 (MOI = 1) for 48 h. The cells were dual-labeled with annexin V and PI and analyzed by flow cytometry. The proportions of apoptotic cells among total cells are expressed as the means ± SEMs of three independent experiments. **(D,E)** MDBK cells were transfected with siRNA or siRNA controls for 24 h, and then infected with CPIV3 (MOI = 0.01). Viral titers were determined by qRT-PCR and TCID_50_ at 24 and 48 hpi. Data are representative of three independent experiments and presented as means ± SDs (*P < 0.05; **P < 0.01).

To examine whether the effects of bta-miR-98 on regulation of apoptosis during CPIV3 infection were mediated through caspase-3, three caspase-3 RNA Interferences (RNAis) were synthesized which could significantly inhibit the expression of caspase-3 at both the mRNA (Figure 8A) and protein levels (Figure 8B). We then examined changes in apoptosis in cells treated with the siRNAs or siRNA controls. Silencing of caspase-3 significantly reduced the percentage of apoptotic cells, demonstrating that caspase-3 silencing had effects similar to those of bta-miR-98 overexpression (Figure 8C). Moreover, CPIV3 replication was significantly suppressed by silencing of caspase-3, similar to the effect of bta-miR-98 overexpression on CPIV3 replication. The viral titers and mRNA levels of CPIV3 were reduced approximately 10-fold compared with controls (Figures 8D,E). These data indicated that bta-miR-98 inhibited apoptosis to suppress CPIV3 replication by targeting caspase-3.

## Discussion

Similar to other *Paramyxiviridae*, CPIV3 has epithelial tropism. We previously demonstrated that CPIV3 could replicate in several types of cell lines, such as peripheral blood mononuclear cells, bronchial epithelial cells, goat kidney cells, and ovine testis cells (OA3.Ts). Nonetheless, most of these cell lines were not susceptible to infection at low viral titers (data not shown). Throughout our study, MDBK cells were used because they are more permissive to CPIV3 infection. Our previous study showed that CPIV3 proliferated stably in MDBK cells and that titers peaked at 10^7^.^0^ TCID_50_/mL at 72 hpi (36). In the present study, approximately 3.0% of apoptotic cells could be detected at 24 hpi, and this figure gradually reached a maximum of 27% at 72 hpi. The increase in apoptotic CPIV3-infected MDBK cells occurred in tandem with time-dependent increases in viral loads. These results indicated that apoptosis in CPIV3-infected MDBK cells may be associated with viral replication.

It was reported that bovine and human parainfluenza viruses type 3 represent a paradigm of the ability of members of the *Respirovirus* genus to trigger apoptosis *in vivo*, as the bronchial epithelium was degenerated and desquamation occurred in challenged animals (37, 38). These results suggested that regulation of apoptosis plays an important role in the replication and pathogenicity of these viruses. Here, we demonstrated that CPIV3 induced apoptosis in MDBK cells. CPIV3-infected MDBK cells exhibited the typical characteristics of apoptosis, including reduction of cell viability and increased rates of apoptosis. However, the precise mechanism involved in CPIV3-induced apoptosis remained elusive. The caspase family included conserved cysteine aspartic-specific proteases, and members of this family are crucial in the regulation of apoptosis during infection by many viruses (39). The extrinsic and intrinsic signals triggered caspase cascades and mediate apoptosis (40). Three important molecules regarded as initiator caspases at the top of the caspases cascades, caspase-8, -9, and -10, were activated, leading to activation of downstream caspases including caspase-3, -6, and -7, and finally resulting in apoptosis (41, 42). In this study, caspase activity assays and western blotting revealed that caspase-3, -8, and -9 activities were increased during CPIV3 infection in a time-dependent manner. Subsequently, increases in the levels of Fas/FasL and the Bax:Bcl-2 ratio in CPIV3-infected cells were detected by qRT-PCR compared with controls. Higher Bax:Bcl-2 ratios were shown to induce release of caspase-9 and -3 (43); meanwhile, regulation of Fas may play an important role in processes (44). Taken together, these data indicated that activation of caspase-8 was trigged by Fas/FasL, while Bax:Bcl-2 regulated apoptosis by activating caspase-9. Thus, CPIV3 infection activated caspase-3 and induced apoptosis via both extrinsic and intrinsic pathways. Moreover, the aberrant miRNAs’ expression profiles of CPIV3-infected cells were reported in our previous study, and bta-miR-98 was predicted as a key regulator involved in apoptosis (33). Thus, it was of interest to evaluate the specific mechanisms underlying the effects of bta-miR-98 on CPIV3-induced apoptosis.

MiR-98, one of the 12 members of the let-7 miRNA family, was first reported to regulate the developmental timing of cell differentiation and proliferation in *C. elegans* (45, 46). A recent study demonstrated that miR-98 was downregulated during infection by various viruses, including hepatitis B virus (47), severe acute respiratory syndrome virus (48), and human adenovirus (49). Consistent with previous studies, bta-miR-98 expression in CPIV3-infected MDBK cells was significantly down-regulated in both a time-dependent and dose-dependent manner (Figure 4). This phenomenon implies that downregulation of bta-miR-98 in CPIV-infected MDBK cells may facilitate CPIV3 replication.

Recently, research has mainly focused on miR-98 regulation of apoptosis and its target proteins including Bcl-2, Fas, caspase-3, and c-Myc (50–53). Expression of miR-98 was downregulated in osteoarthritis (OA) cartilage tissues. In a rat model of OA, exogenous injection of miR-98 mimics inhibited cartilage cells apoptosis, thus alleviating OA (54). Therefore, miR-98 miRNAs are considered as important in regulating cell cycle progression and apoptosis. Here, we demonstrated a mechanism for this effect in which bta-miR-98 negatively regulated apoptosis by targeting the 3′-UTR of caspase-3 mRNA. We concluded that CPIV3 infection downregulated bta-miR-98 expression to promote apoptosis and facilitate its replication.

Caspase-3, -6, and -7 are important for the execution of certain downstream events in apoptosis. Limited information is available on the roles of the other two effector caspases, caspase-6 and -7, but these may lack significant specialized functions (55). Analysis of apoptosis progression in caspase-3 knockout animals revealed complete inhibition of membrane blebbing, DNA degradation, and nuclear fragmentation (56, 57). Moreover, caspase-3 was demonstrated to be crucial for poly (ADP-ribose) polymerase (PARP) cleavage and DNA fragmentation, which are regarded as apoptotic hallmarks (58). In this study, expression of caspase-3 was significantly upregulated, while levels of bta-miR-98 were markedly reduced in CPIV3-infected cells. These results implied that bta-miR-98 was hijacked by CPIV3 to increase caspase-3 activity. To demonstrate interaction of bta-miR-98 with the 3′-UTR of caspase-3, we performed a dual-luciferase reporter assay and observed that 293T cells co-transfected with bta-miR-98 mimics and a caspase-3 whose 3′-UTR carried a bta-miR-98 binding site significantly suppressed luciferase activity. By contrast, bta-miR-98-mut had no effect on luciferase activity. These data indicated that bta-miR-98 targets the 3′-UTR of caspase-3, and that caspase-3 is negatively regulated by bta-miR-98 during CPIV3 infection. In addition, bta-miR-98 mimics significantly decreased the percentage of apoptotic cells and the activities of caspase-3, -8, and -9 to suppress CPIV3 replication, while bta-miR-98 inhibitors had the opposite effect (Figure 5). Silencing of caspase-3 restored the inhibitory effect of bta-miR-98 mimics on CPIV3-induced apoptosis and reduction of viral replication. Taken together, our findings demonstrate that bta-miR-98 negatively regulated CPIV3-induced apoptosis by targeting caspase-3 and suppressed replication of CPIV3.

**FIGURE 9:**
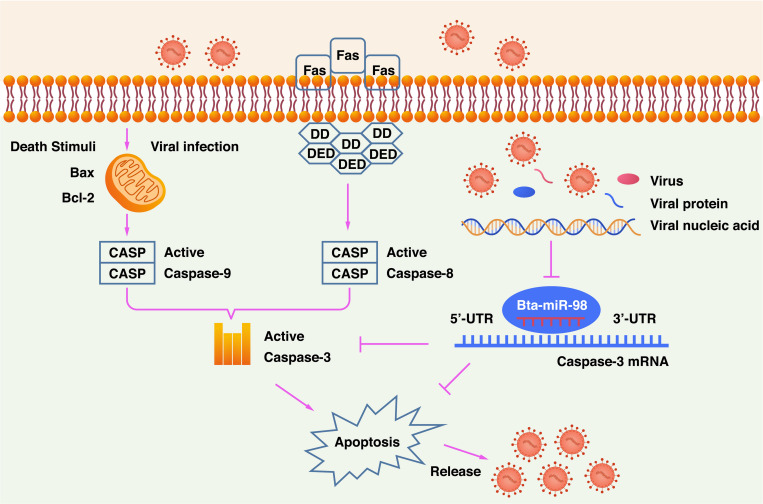
Schematic diagram of bta-miR-98 inhibition of apoptosis for suppression of CPIV3 replication. CPIV3 induces apoptosis through both extrinsic and intrinsic pathways and suppresses bta-miR-98 expression. Bta-miR-98 can inhibit apoptosis by targeting caspase-3 and suppresses CPIV3 replication.

In summary, this study provides evidence that CPIV3 induced apoptosis by activating caspase- 3-, - 8-, and -9-mediated apoptotic signaling. Additionally, bta-miR-98 acted as a negative regulator of CPIV3-induced apoptosis. Overexpression of bta-miR-98 inhibited caspase-3 signaling, resulting in decreased apoptosis and suppressing CPIV3 replication (Figure 9). To the best of our knowledge, this is the first report showing that bta-miR-98 mediated regulation of caspase-3, meaning it may participate in the induction of apoptosis and viral replication. Therefore, our results may provide insight into the use of bta-miR-98-based therapeutics against apoptosis-induced diseases.

## Data Availability Statement

The datasets generated for this study can be found in the GenBank accession no. NC_028362.1, miRBase no. MI0005025.

## Author Contributions

JL took part in all the experiments and wrote the manuscript. XJ, ML, and CL helped to design the whole project and draft the manuscript. CZ, ZL, LM, and WL conducted RNA isolation and sample processing for sequencing. MS, TX, LY, and WZ conducted data analysis. JL, MS, and LM provided experiment materials. All authors read and approved the final manuscript.

## Conflict of Interest

The authors declare that the research was conducted in the absence of any commercial or financial relationships that could be construed as a potential conflict of interest.
